# European Collaboration in Health Technology Assessment (HTA): goals, methods and outcomes with specific focus on medical devices

**DOI:** 10.1007/s10354-019-0684-0

**Published:** 2019-02-06

**Authors:** Judit Erdös, Sabine Ettinger, Julia Mayer-Ferbas, Cecilia de Villiers, Claudia Wild

**Affiliations:** Ludwig Boltzmann Institute for Health Technology Assessment, Garnisongasse 7/20, 1090 Vienna, Austria

**Keywords:** European Network for Health Technology Assessment, EUnetHTA, Cross-border health care, European Union, Europäisches Netzwerk für Health Technology Assessment, EUnetHTA, Grenzüberschreitende Gesundheitsversorgung, Europäische Zusammenarbeit

## Abstract

The European Network for Health Technology Assessment (EUnetHTA) was founded to support efficient production and use of health technology assessments (HTAs) across Europe by reducing redundancies through collaboration. To facilitate collaboration, a range of practical tools, methods and process definitions were developed. The article describes when and how these tools and methods are used along the HTA process with specific focus on “other technologies”, that is medical devices and non-pharmaceutical procedures. EUnetHTA was able to deliver tangible achievements complying with its goals. The practical tools and the developed methods formed a basis for close collaboration among over 70 agencies at a European level. The activities of EUnetHTA laid a strong foundation for sustainable cooperation. In the long run, jointly produced assessments could realise economies of scale with improved quality, consistency and transparency for the health systems in Europe.

## Zusammenfassung

Das Europäische Netzwerk für Health Technology Assessment (EUnetHTA) verfolgt das Ziel, mittels Zusammenarbeit eine effiziente Erarbeitung und Anwendung von HTA in europäischen Ländern zu unterstützen und Redundanzen zu vermindern. Um diese Zusammenarbeit zu erleichtern, wurden eine Reihe praktischer Tools, Methoden und Prozessdefinitionen entwickelt. Im vorliegenden Artikel wird beschrieben, wann und wie diese Tools und Methoden im HTA-Prozess verwendet werden, wobei der Fokus auf „other technologies“ liegt, d. h. Medizinprodukte und nichtpharmazeutische Verfahren. Dabei konnte das EUnetHTA bereits konkrete Erfolge in der Erreichung der Ziele verbuchen. Die praktischen Tools und Methoden stellen eine Grundlage für die enge Zusammenarbeit von über 70 Agenturen auf europäischer Ebene dar. Durch die Aktivitäten des EUnetHTA wurde eine bedeutende Basis für eine nachhaltige Zusammenarbeit geschaffen. Gemeinsam durchgeführte Bewertungen können langfristig zu Skaleneffekten, höherer Qualität und verbesserter Transparenz für die Gesundheitssysteme in Europa führen.

## Introduction

All healthcare systems in Europe and worldwide have to make decisions on investments in new medical interventions such as screening programs, large technical equipment, drugs or medical devices. The work programmes of European health technology assessment (HTA) agencies therefore comprise similar topics, as decision support for such investments is often given at the same point in time. This insight is not novel and is mainly based on an overview of the HTA database for published reports [1]. To avoid the apparent duplication and the inefficiency of conducting several similar HTAs globally, the HTA research community launched this initiative in the early 90s to harmonise methodologies and coordinate collaboration. The European Network for Health Technology Assessment (EUnetHTA) started in 2006 and is still supported by European Commission research grants. The aim was to establish a network of public national HTA agencies, research institutes and health ministries. The project was followed by a transition year before the first joint action (JA) started in 2010. The Cross Border Directive (2011) provided the political and regulatory framework for Joint Action 1 (JA1) and the succeeding JA2 and JA3, stating in article 15 that “The Union shall support and facilitate cooperation and the exchange of scientific information among Member States within a voluntary network connecting national authorities or bodies responsible for health technology assessment designated by the Member States” [2].

The third JA (JA3) period is currently ongoing (2016–2020). The mission of EUnetHTA is to support efficient production and use of HTA in countries across Europe through the reduction of redundancies and duplication of effort, and by strengthening the link between HTA and healthcare policy making. The objectives evolved through the project itself and the subsequent JAs. In JA1 the main objective was to enable an effective exchange of information and support of policy decisions. In JA2 the focus was laid on establishing an effective and sustainable HTA collaboration in Europe and strengthening the practical application of tools and approaches to cross-border HTA collaboration. Finally, in JA3 the goal is mainly to define and implement a sustainable model for European cooperation on HTA^1^ post 2020 [3, 4].

In EUnetHTA the life cycle concept of health technologies is followed. It starts with early dialogues (ED) intending to advise the manufacturer of drugs and devices on study designs, comparators and outcomes relevant for HTA. It spans from early pre-coverage assessments, including rapid relative effectiveness assessment (REA) of a single technology or a bundle of technologies and procedures, to later post-coverage updates, as well as post-launch additional evidence generation [5]. One of the major challenges that calls for action by EUnetHTA is the reduction of duplication, both in terms of assessment production and evidence generation. In the current JA3, the work is organised into seven work packages (WP), from which two separate work packages are dedicated to these two areas of action. WP4 is responsible for the coordination of collaborations in producing assessments and WP5 is responsible for pre- and post-market authorization evidence generation.

This article aims to outline the goals, the applied methods and the achieved results of collaboration at a European level in HTA. There is a dedicated focus on a specific part of the joint work, which is the collaborative production of assessments of medical devices (mainly risk class IIb and III) and procedures. In EUnetHTA, these non-pharmaceutical interventions are called “other technologies” (OT), and their production is managed by the Ludwig Boltzmann Institute for Health Technology Assessment (LBI-HTA) in Austria.

## Materials and methods

The following tools, methods and processes have been developed since 2006 to facilitate collaboration.

- Topic identification, selection and prioritization process (in development)
- Planned and Ongoing Projects (POP) Database
- Internal procedure manual describing the process of project management of EUnetHTA assessments and European Activity Centres for managing collaborative assessments
- Procedure manual for rapid relative effectiveness assessment (REA)
- Templates: project plan template, assessment template, evidence submission template
- Standardized reporting structure: the CoreModel®
- EUnetHTA methodological guidelines
- Processes of stakeholder involvement (in development)
- Evidence database on new technologies (EVIDENT database)
- Companion Guide comprising all templates, guidelines and process descriptions in the format of standard operating procedures (in development)
- EUnetHTA website

**Fig. 1:**
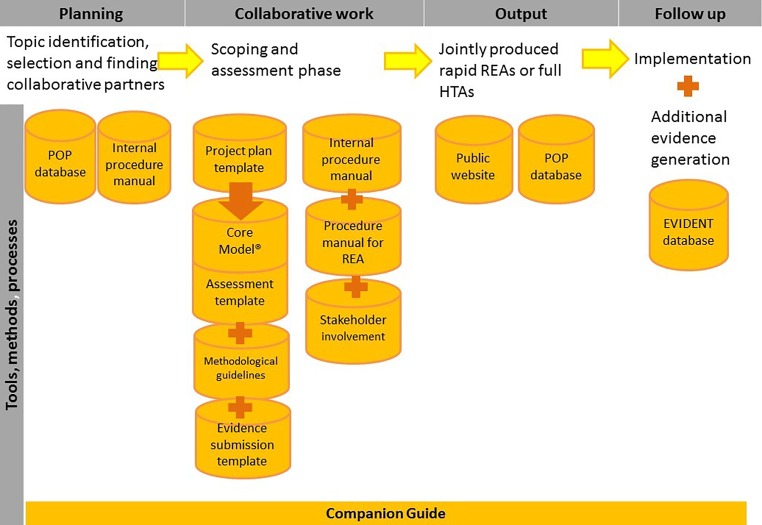
EUnetHTA tools and methods in the process of the production of an assessment. *POP database* Planned and Ongoing Projects Database, *REAs* Relative Effectiveness Assessments, *EVIDENT database* Evidence Database on New Technologies

We describe step-by-step when and how these tools are used in the assessment process with a specific focus on OT (Fig. 1) and discuss experiences with them. We also present some real-life examples to show the uptake of EUnetHTA assessments. Finally, we address some challenges EUnetHTA faces and the outlook for the post-2020 period.

## Results

### 1. Planning:

The first step in starting joint work on assessments is finding the topic to work on and or the partners to work with.

#### Topic identification, selection and prioritization process

A process for topic identification, selection and prioritization for EUnetHTA assessments is under development and is expected to be presented to the public in 2019. The current practice, as described below, remains in place until the recommendations by the task group are implemented. Topics concerning assessments of medical devices and procedures are currently identified in three ways [6]:

- A EUnetHTA partner selects a topic from their national work programme and a call for collaboration is sent to the other partners. If the topic is relevant to other partners, then they are invited to volunteer for the role of co-author or reviewer. If there is no interest in the topic, the assessment will not be performed as a EUnetHTA assessment, but could be performed at a national level by the individual partner. The process is managed centrally by LBI-HTA, the leading partner on “other technologies” in WP4.
- Topics are suggested by stakeholders (restricted to manufacturers and/or patient organisations), and a call for collaboration is sent asking for authors, co-authors and reviewers. If there is no interest in the topic, the assessment will not be performed. The process is managed centrally by LBI-HTA.
- A EUnetHTA partner identifies a topic in the POP database (please see description of the database below) that is identical to the topic they are planning to work on and contacts the partner which has the entry in the database to enquire about possible collaboration. If the partner agrees on a collaboration and on their role (co-author or dedicated reviewer), a call amongst EUnetHTA partners can be sent out for the remaining roles of co-authors or reviewers.

The majority of OT prioritised for assessment have so far been identified by the first route [6].

#### Planned and Ongoing Projects (POP) database

The POP database allows EUnetHTA partners to share information with each other on planned, ongoing or recently published projects conducted at the individual agency. The aim of the database is to reduce duplication and facilitate collaboration among EUnetHTA partners. The rate of similar or identical projects has continuously been around ten percent since the start of JA1 [7]. A thorough evaluation of the usage of the database at the end of JA1 [8] reported that 75% of the replying partners considered the POP Database a useful tool for sharing knowledge, experience and information. 42% indicated that the POP database provided an important first-hand overview of ongoing HTA activities at a European level as well as an easy access to other agencies. 17% stated that they had the impression that collaboration had increased efficiency. A direct reduction of duplication in conducting HTAs was reported twice. Collaborative activities usually focus on information exchange of literature search protocols, extraction tables, information on the description and the technical characteristics of the technology as well as the health problem, executive summaries and full project reports [8].

The limited feedback from database users makes it difficult to show the precise number of collaborations initiated from the database information. Based on the available information from survey questionnaires conducted in JA2 and personal communication, the practice of deferral of an assessment started to form. This means that the agencies wait for the other agency to finish a project before they start their own one when they find the same topic in the POP database. Therefore, the current value of the database lies mainly in its ability to inform the EUnetHTA partners about what other EUnetHTA partners are working on and thus save resources by waiting for their results or exchanging the project plan, search strategies and search results, extraction tables, etc. This is also a mode of reduction of duplication. The POP database’s role as a facilitator of collaboration in the sense of producing joint reports is yet to be strengthened.

### 2. Scoping and assessment phase

#### Internal procedure manual describing the process of project management of EUnetHTA assessments

**Fig. 2:**
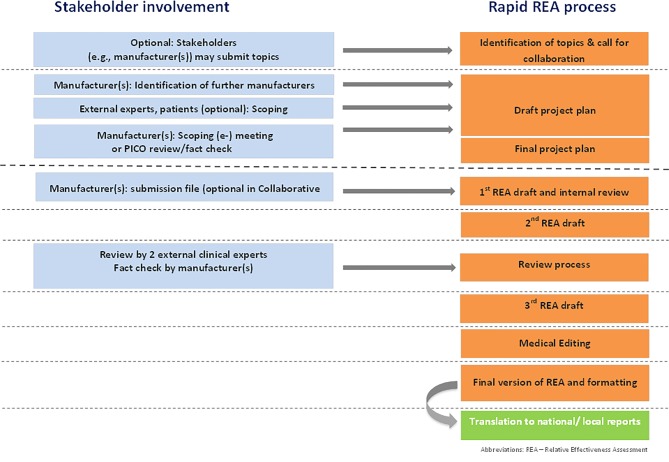
Production process in “other technologies” (medical devices, procedures) [9]

The project management is an overarching activity in the production of an assessment (Fig. 2). An internal procedure manual, aimed at supporting project managers, started to evolve during JA2 and has been a dynamic document ever since. It reflects the developments and changes during JA2 and JA3, and is gradually being transformed into standard operating procedures (SOPs) in cooperation with WP6, which is responsible for quality management, scientific guidance and tools. The internal procedure manual is not available to the public.

#### European Activity Centres for managing collaborative assessments on other technologies

**Fig. 3:**
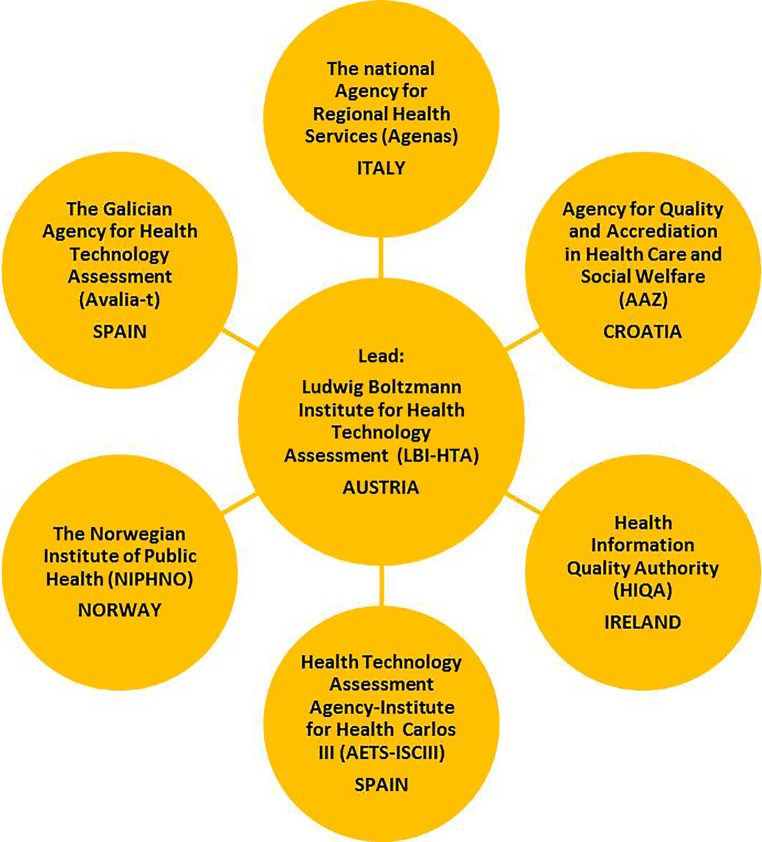
Activity Centres for managing collaborative assessments of “other technologies”

In order to achieve the general objectives of JA3, two modes of project management are piloted. A centralized mode of project management for jointly produced assessments of pharmaceuticals and a decentralized mode for other technologies (OT). The centralized mode for pharmaceuticals results from the fact that all new medicines go through a centralized authorization procedure at the European Medicines Agency (EMA). On the other hand, OT enter the European healthcare systems at different points in time, and therefore a phased roll-out of the project management activities from a centralized into a decentralized form was started and is being explored. Six EUnetHTA partners, all experienced in the assessment of medical devices and procedures, volunteered to become Activity Centres for European collaborative assessments on other technologies, resulting in managing their own assessments and, if required, assessments of other partners. LBI-HTA manages the topic identification, selection and prioritization process, including the call for collaboration centrally, and supervises their activities as well as providing support and training (Fig. 3). Within the first 2 years of JA3, around 20 jointly produced assessments on other technologies were either published or are ongoing.

#### Procedure manual for rapid relative effectiveness assessment of other technologies

The procedure manual for relative effectiveness assessment (REA) of other technologies [9] bindingly guides authors, co-authors, dedicated reviewers and project managers in the production process of EUnetHTA assessments. The standardization helps to keep the production process transparent and uniform. The manual contains a detailed description of the assessment stages, including naming the tools, templates and guidelines to be used and guidance on their use. Project management-related issues, like composition of the team, and communication are also addressed. All the information that is covered in the procedure manual will be transformed into SOPs by the end of JA3, in the same way as for the internal procedure manual.

#### Standardized reporting structure: the CoreModel®

The HTA CoreModel® is a methodological framework for production and sharing of HTA information. The model consists of the following three components, each with a specific purpose [10–13]:

- A standardised set of HTA questions allowing users to define their specific research questions within a hierarchical structure.
- Methodological guidance to assist in answering the research questions.
- A common reporting structure for presenting findings in a standardised question–answer pair format.

The CoreModel® was originally developed within the EUnetHTA project in 2006–2008 and further improved during JA1. It is currently under revision and an updated model is expected to be delivered by the end of JA3. There are different CoreModel® versions for the production of Core HTAs containing all nine domains of a comprehensive HTA: 1. Health problem and current use of the technology, 2. Description and technical characteristics, 3. Safety, 4. Clinical effectiveness, 5. Costs and economic evaluation, 6. Ethical analysis, 7. Organisational aspects, 8. Patient and social aspects, 9. Legal aspects. The production of rapid REAs, which is the type of assessment that is produced in JA3, contains only a subset of domains [13]. Each domain is further divided into a set of generic research questions to be considered. When using the Model, the generic questions are transformed into actual topic-related research questions. The aim is that information can easily be located and shared due to the transparent way of reporting, therefore easing the cooperative work. The Model contains no recommendations, but only the factual information and evidence required to make a recommendation [11, 12].

#### Templates in the production process

Several templates for standardized steps in the process of the production of jointly produced assessments have been developed:

- Project plan template and related comments forms for internal and external review.
- Assessment template and related comments forms for internal and external review: the assessment template is based on the Core Model® common reporting structure and is used in all EUnetHTA assessments. The template was developed as an online tool, but was then substituted by a simple word document after pilot testing.
- Evidence submission template: a tool that can be used by national agencies as well as in EUnetHTA to request evidence from companies to support their HTA and reimbursement processes. Evidence requirements for reimbursement in Europe were analysed and synthesized to create the tool. It follows the same structure as relative effectiveness assessments, including a description of the health condition and health technology, as well as clinical effectiveness and safety information [14, 15].

#### Methodological guidelines

The primary objective of EUnetHTA methodological guidelines is to focus on challenges that are encountered by HTA assessors while performing relative effectiveness assessments of pharmaceutical or other technologies. Examples are a differentiation between surrogate and patient-relevant endpoints, validated methodologies measuring quality of life or guidance in assessing medical devices. A number of guidelines were developed in JA1 and JA2 and are being revised in JA2 and JA3 [16], but also a number of new guidelines are in the pipeline. For example, the Critical assessment of clinical evaluations and Critical assessment of economic evaluations [16]. All guidelines are publicly available on the EUnetHTA website [16] and the use of the guidelines is mandatory in the EUnetHTA assessments.

#### Stakeholder involvement

Stakeholder involvement in EUnetHTA is necessary to ensure the legitimacy of EUnetHTA and its products. The stakeholders are defined as regulators, payers and policy-makers, research and academia, industry, patients and consumers, healthcare providers (including healthcare professionals and hospitals) and HTA organisations outside of EUnetHTA or Europe [17]. The representation of interests is encouraged, thus contributing and promoting the utilization of HTA in national and regional policy making [4]. In JA1, a policy [18] and a SOP [19] on stakeholder and expert involvement [20] were developed and remained in use during JA2 and JA3. The policy set the aim of establishing fair opportunities to provide input for all stakeholders while ensuring scientific independence from undue influence of external parties.

In JA3 interactions with stakeholders occur on different levels, namely task-specific activities or horizontal activities. Horizontal activities include participation in the EUnetHTA Forum, a platform for network-wide scientific discussions and exchange of experience between the members of EUnetHTA JA3 and interested stakeholders. Task-specific activities in WP4 relate to patients or consumers, payers and regulators, healthcare providers and industry. The stakeholders are invited to provide input in the production process of jointly produced assessments and/or their implementation. The different stakeholder groups can be involved in various stages of the assessment production, for example in reviewing and commenting on the project plan, and the methods of their involvement are manifold.

If patient involvement is planned, patient organisations and or individual patients are contacted to provide input in the scoping phase of the assessment. Patient groups and their caregivers can help understand patients’ unique perspectives. Recommendations on preferred methods for collecting patient input are currently developed by a task group. So far, out of twenty EUnetHTA assessments of other technologies, eight applied any kind of patient involvement. The methods used and tested by the authoring teams of the assessments that involved patients are very diverse: focus group meetings for identifying patient-relevant endpoints [21], written feedback by individual patients on the population-indication-comparator-outcomes (PICO) or semi-structured interviews with individual patients, scoping e‑meeting for discussing PICO and collection of patient input using a modification of the HTAi Patient Group Submission template [22].

It is a mandatory step to involve healthcare providers (clinicians) in the scoping and in the review phase of the assessment. At least two external clinical experts review the draft project plan and the draft assessment before publication. During scoping the experts are consulted for their comments on the PICO. They submit their comments on a standardised comments form which is published along with the project plan and the authors’ replies to the comments. The same process is followed for the draft assessment. The experts are also asked to participate in the scoping (e-)meeting to discuss issues raised by them and uncertainties raised by the assessment team. In JA3 the methods of involvement of healthcare providers are being discussed and redefined.

Industry involvement can take various forms. Manufacturers can submit topics which they deem relevant for assessment at a European level to EUnetHTA. All partners are notified of these topics. The assessment is performed only if there is interest among the partners and a sufficient number of partners volunteer. Once an assessment has started, the manufacturer(s) of the respective technology is (are) contacted and offered the opportunity to comment on the preliminary PICO. During the scoping phase they can be invited to a face-to-face or an e‑meeting. Formal input can be requested using an evidence submission file [14, 15] and/or asking for a final factual accuracy check during the external review of the assessment.

#### The Companion Guide

Every process described in the planning, scoping and assessment phases will be covered by standard operating procedures (SOPs) and be integrated into the Companion Guide. This will contain all SOPs, templates and methodological guidelines as well as the practical tools (the CoreModel®, the POP database and the EVIDENT database) for HTA assessors [23]. The users have the option to either see the whole list of SOPs or select their role in the assessment (author, reviewer, project manager) and sort the SOPs accordingly. The Companion Guide will be publicly available after JA3.

### 3. Follow-up

#### Implementation

After finalisation of the assessment, the report is disseminated broadly inside and outside of the European network for HTA. Partners can use the report directly without making any changes or adapt it to their national needs. The report or only the executive summary can be translated into the native national language. Amendments can be made or it can be complimented with country-specific information including reimbursement information, epidemiological information, organisational or legal issues. The purpose of adaptation is to enable an HTA agency in one setting to make use of an HTA report produced elsewhere, thus saving time and resources [24].

National uptake of HTAs is one of the main goals in JA3 to increase the use, quality and efficiency of joint HTA work at the European level. EUnetHTA supports re-use in regional and national HTA reports and activities. The re-use is measured and monitored, and the information is collected via feedback surveys and complemented by interviews conducted by WP7 [25]. Detailed data are available with a certain time lag, since not all assessments are re-used immediately after their publication. Results of re-use and uptake are presented in the JA3 implementation report [25]. The response rate to the survey regarding JA3 OT assessments ranged from 66 to 71%. The use of the OT assessments was reported in 25 cases and can be categorized into two types:

- Supporting the agency’s existing HTA activities or serving as their alternative (substitution: 44%). The EUnetHTA assessments were most commonly used to inform an agency procedure for reimbursement.
- Dissemination to raise awareness of EUnetHTA assessments and or evidence informed decision-making (complementary: 56%).

The implementation report [25] identified some barriers to re-use of EUnetHTA assessments, such as timing constraints due to the EUnetHTA assessments still in progress at the time of the policy request for a national assessment or EUnetHTA assessments not being up-to-date. Another reason might be the different scope of the EUnetHTA assessment from that of the agencies. The reporting structure used in EUnetHTA and the language needs in national practice are further hindrances. Approximately 4 months of staff-time are the average efficiency gains for agencies re-using EUnetHTA reports. In most cases the agency conducted only translation or in some instances local information was added.

#### Additional evidence generation

Once the authors of an assessment have noted evidence gaps, these can not only be mentioned in the assessment, but are collected in a central database, the Evidence Database on New Technologies (EVIDENT Database). This is a tool to promote generation of additional evidence and facilitate European collaboration in the area of post-launch evidence generation. The database allows sharing and storage of information on:

- Reimbursement or coverage and assessment status of promising technologies and
- Requests or recommendations for additional studies arising from HTA.

If EUnetHTA partners contribute relevant, accurate and timely information to the database, and use it regularly, an overview of the coverage and marketing approvals of a certain technology could be achieved at a European level [10]. The EVIDENT Database has not been used extensively so far for these purposes.

## Discussion

Networks of collaboration in HTA have a long history dating back to the late 90s. None of them came as far as EUnetHTA in their activities aimed at harmonization of methods as the basis for collaboration. EUnetHTA, backed by the regulatory framework of the Cross Border Directive [2], could achieve the main objectives outlined at the beginning of each joint action and delivered tangible achievements complying with the goals that were set. The practical tools and the developed methods formed a basis for close collaboration among over 80 agencies across Europe. The benefits of collaboration such as shared knowledge on planned and ongoing projects elsewhere, improved methodological quality, standardisation of practices and processes, and capacity building in countries with less experience in conducting HTAs reach beyond Europe and also have an impact internationally. Understandably, smaller countries, such as Austria, with fewer resources available to cover a wide range of technologies, are more eager to collaborate compared to larger countries. The resultant resources available could then be utilized to expand local services to the national decision-makers.

The identified challenges and shortcomings in EUnetHTA need to be addressed during JA3 in preparation for a sustainable network post 2020. Duplication is still present to some extent and can be attributed to various reasons. Practical barriers such as language use, reporting structure, and the differences in national processes and methodologies, including the timing and scope of the assessments, can contribute to redundant HTA products. These barriers need to be solved in the long run to ensure the objectives of resource savings and avoiding redundancy. Those agencies using EUnetHTA assessments instead of conducting their own assessments could significantly benefit from the time-savings. In order to maximize value and increase efficiency gains for EUnetHTA partners, most importantly the timing, the topic selection, the scope and the quality of the assessment need to be aligned to ensure that the assessment is applicable for as many partners as possible. The permanent cooperation after 2020 would potentially solve some of the mentioned challenges.

In January 2018, the European Commission (EC) published the “Proposal for a Regulation of the European Parliament and of the Council on health technology assessment and amending Directive 2011/24/EU” [26]. The proposal has the specific objectives “to promote convergence in HTA tools, procedures and methodologies; to ensure efficient use of resources and strengthen the quality of HTA across the EU and to improve business predictability.” This proposal is based on intensive mapping of HTA structures and methodologies [27, 28] and impact analyses [29] of all member states and can be interpreted as a sign of commitment and a strong wish from all parties to strengthen the cooperation and not lose the achieved results. The ultimate goal is that the developed methods, tools and processes will become established and remain in place in a sustainable (EC-supported, but member states-driven) network. The proposal, aiming for adoption by 2019, is now under consultation and discussion in the European Parliament and the Council. The policy options described in the impact analysis [29] range from a voluntary cooperation (option 1) to a permanent cooperation on common tools, methodologies, early dialogues and joint relative effectiveness assessments with a mandatory national uptake of the joint clinical assessments (option 4). The latter option is the preferred option according to the analysis conducted by the European Commission.

## Conclusion

The activities of EUnetHTA, co-funded by the European Commission, has brought the collaboration of European HTA agencies to another level and laid a strong foundation for a sustainable cooperation. If the proposal is accepted in its current form, the duplication might be addressed, and national uptake might increase considerably. In the long run, jointly produced assessments would realise economies of scale with improved quality, consistency and transparency for the health systems in Europe.

## Footnotes

1 European Commission Consumers, Health, Agriculture and Food Executive Agency (CHAFEA). Grant Agreement Annex 1—Description of the Action (Number 724130—EUnetHTA JA3).
